# Changes in gut microbiota in *Gynura segetum*-induced liver injury

**DOI:** 10.3389/fmicb.2025.1684570

**Published:** 2025-11-26

**Authors:** Menghui Zhang, Lu Liu, Jianchun Lian, Minna Zhang, Xiaozhong Yang, Honggang Wang

**Affiliations:** 1 Department of Gastroenterology, The Affiliated Huaian No. 1 People's Hospital of Nanjing Medical University, Huai'an, Jiangsu, China; 2 Department of Pathology, The Affiliated Huaian No. 1 People's Hospital of Nanjing Medical University, Huai'an, Jiangsu, China; 3 Laboratory Department, The Affiliated Huaian No. 1 People's Hospital of Nanjing Medical University, Huai'an, Jiangsu, China

**Keywords:** intestinal flora, hepatotoxicity, *Gynura segetum*, tusanqi, fecal microbiota transplantation

## Abstract

**Introduction:**

*Gynura segetum* (GS) has been shown to induce hepatotoxicity. Growing evidence suggests that the response to herbal medicines may be linked to shifts in the gut microbiota. This study aims to investigate the association between gut microbiota and liver injury induced by GS.

**Methods:**

The mice model of liver injury was established by oral gavage of GS decoction for 4 weeks, with or without the broad-spectrum antibiotics (Abx) or fecal microbiota transplantation (FMT). Liver function was assessed through the hematoxylin and eosin (H&E) staining and biochemical indices. The microbiota in the intestinal tract and peritoneal cavity were determined by 16S rRNA gene sequencing. Senecionine, seneciphylline, ferulic acid, beta-sitosterol, vanillic acid, vanillin, isorhamnetin, quercetin, kaempferol, and luteolin were isolated from GS plants, and the effects of these chemical compounds on the intestinal flora were analyzed.

**Results and discussion:**

Compared to controls, mice treated with the GS decoction exhibited decreased body weight and increased serum levels of total bilirubin, direct bilirubin, alanine aminotransferase, and aspartate aminotransferase, regardless of whether they were given Abx or FMT. The abundance of *Akkermansia* (phylum *Verrucomicrobia*) persistently increased in the GS group. In contrast, other bacterial groups showed different trends under Abx or FMT conditions. Additionally, compared with the GS group, the linear discriminant analysis (LDA) score revealed the increase in abundance of *Bifidobacterium, Bacteroides, Ruminococcaceae_UCG-007*, and *Coriobacteriaceae_UCG-002* in the Abx group, and *Blautia* and *Bifidobacterium* in the FMT group. 16S sequencing of ascitic fluid detected multiple bacterial phyla. Moreover, the administration of chemical compounds isolated from the GS plant by gavage did not increase the abundance of *Akkermansia* in the intestine.

**Conclusion:**

GS increased the relative abundance of the *Akkermansia* genus in the intestinal tract. None of the above chemical compounds had this effect. This suggests that some components of GS may promote the growth of beneficial bacteria such as *Akkermansia*, offering new perspectives for drug development.

## 1 Introduction

Resident bacteria in the human intestine play a crucial role in nutrient metabolism, immune defense, and maintaining intestinal ecology. Taxonomic variations in the aggregate flora of an individual are rather narrow, and marked perturbations, such as diet, infection, or antibiotics, can cause instantaneous dysbiosis or even illness. The intestinal flora interacts with multiple systems throughout the body, and its imbalance has been linked to a range of conditions, such as digestive, cardiovascular, mental, neurological, liver, chronic nephritic, malignant, autoimmune, and inflammatory rheumatic diseases (Uchiyama et al., 2019; Gowen et al., 2023; Hsu and Schnabl, 2023). The causal relationship between the disease phenotype and specific microorganisms is currently under investigation. Therapies that target intestinal bacteria, including probiotics, prebiotics, synbiotics, fecal microbiota transplantation (FMT), and dietary alterations, have been used clinically as an effective treatment of above diseases (Sadeghi et al., 2023).

The World Health Organization reports that 80% of the population in developing countries still relies on herbal medicines for healthcare, with artemisinin for malaria being a prime example (Talman et al., 2019). Recent research indicates that herbal medicines (HMs) can alter the composition of the intestinal microbiota, which may help improve or treat conditions such as metabolic diseases, inflammatory bowel disease (IBD), psoriasis, malignancies, chronic kidney disease, and Alzheimer's disease (Wang et al., 2023). In addition, microorganisms encode many types of enzymes, which can catalyze biotransformation and metabolic reactions of the HM components, modifying their bioavailability or toxicity. Herbal-medicine therapies act through synergistic or antagonistic interactions with the gut microbiota.

*Gynura segetum* (GS), or TuSanqi, frequently mistaken for Sanqi, is commonly consumed for its hemostatic properties and ability to relieve pain and swelling. However, its consumption has been associated with liver toxicity, largely due to pyrrolizidine alkaloids (PAs). While originally thought to be harmless, PAs are metabolized in the liver into toxic compounds that can cause liver damage through various mechanisms. PAs were metabolized by the liver cytochrome P450 to form toxic dehydropyrrolizidine alkaloids (DHPAs) and dehydroretronecine (DHR). The putative pathogenetic mechanisms of DHPAs and DHR involve (Ma et al., 2018; Zhu et al., 2021): (1) covalent bonding with proteins, RNA, and DNA, which may affect protein synthesis and normal nucleic acid metabolism in the organism; (2) acting as a hapten causing immune-mediated toxicity; (3) decreasing the level of glutathione and inducing of lipid peroxidation and hepatocyte apoptosis. PA-containing plants are distributed worldwide, and cases of PA poisoning are frequent in China. Considering the high mortality rate of this disease and the lack of effective therapies, we have focused on the association between microbiota and HMs associations. Wang et al. established a hepatic sinusoidal obstruction syndrome (HSOS) mouse model through the administration of monocrotaline and demonstrated that oral antibiotic treatment could reverse liver injury (Xiao et al., 2022). However, they did not further investigate whether fecal microbiota transplantation (FMT) could similarly reverse hepatic damage. In contrast, a study by Zhao and co-workers reported that FMT in HSOS-model mice aggravated liver injury (Zhao et al., 2024), a finding that contradicts recent evidence suggesting that FMT can be therapeutic in other disease settings. The objective was to evaluate the effect of GS exposure on gut microbiota, and to determine whether the elimination or modification of the gut microbiota could mitigate GS-induced liver disease. This study also aimed to explore chemical substances that could induce alterations in the gut microbiota.

## 2 Materials and methods

### 2.1 Preparation of the *Gynura segetum* decoction

*Gynura segetum* samples were collected in Bozhou, Anhui province, China. The extraction of the medicinal ingredients followed the traditional Chinese medicine decoction method. The dried root of GS (1000g) was crushed and soaked in water for 2 h. The GS decoction was decocted twice, 2 h each time, and the obtained filtrates were combined and concentrated to 1000 ml. The concentration of the crude drug was 1g/mL.

### 2.2 Animal experiments and specimen collection

Female KM mice (8 week old) were purchased from Cavens Laboratory Animal Co. Ltd. The mice were housed in the animal room at 22-25 °C, 45% to 70% humidity, 12/12h light-dark cycle, and diet and water ad libitum. All procedures were performed in accordance with the Guideline for the Care of Laboratory Animals published by the US National Institutes of Health (NIH Publication No. 85-23, revised 1996), and the protocols were approved by the Animal Care and Use Committee of the Affiliated Huaian No.1 People's Hospital of Nanjing Medical University (Approval No: DW-P-2024-026-01).

The mice were randomly divided into four groups (*n* = 3 per group): control, GS, antibiotics (Abx), and fecal microbiota transplantation (FMT). Control mice were given normal saline (30 mL/kg, once daily), and the remaining groups were gavaged with GS decoction (30 g/kg) daily for 4 weeks. Mice in the Abx group were treated with a cocktail of antibiotics every 12 h for 7 days prior to GS exposure. The antibiotics cocktail consisted of ampicillin (100 mg/kg, Amresco Inc.), vancomycin (50 mg/kg, Amresco Inc.), metronidazole (100 mg/kg, MP Biomedicals), and neomycin (100 mg/kg, Amresco Inc.) (Zarrinpar et al., 2018). Mice in the FMT groups were gavaged every three days with a fecal slurry (30 mg per mouse). To obtain the fecal slurry, fresh feces of control mice were collected and suspended in PBS (50 mg/ml) by vortexing. Major components of GS are alkaloids, terpenes, flavonoids, and other active ingredients (Zhou et al., 2020). To identify the effects of structurally different compounds on the intestinal flora, mice were randomly distributed into groups treated for 4 weeks by seneciphylline (Phy), ferulic acid (FA), beta-sitosterol (BS), vanillic acid (VA), vanillin (Van), isorhamnetin (Iso), quercetin (Que), kaempferol (Kae), luteolin (Lut) (all >98% purity, Chengdu Biopurify Phytochemicals Ltd.), and normal saline (control group). These compounds were administered intragastrically at a dose of 20 mg/kg body weight. An additional group consisted of mice treated with a toxic dose of senecionine (Sen) (>98% purity, Chengdu Biopurify Phytochemicals Ltd.), 10 mg/kg body weight for 28 day. The body weight, appearance of body hair, activity, abdomen changes, and appetite were monitored daily. Fresh feces were collected at 0, 1, 2, 3, and 4 weeks and stored at−80 °C until analysis. At the end of the experiment, blood samples were collected, allowed to stand for 3 h, centrifuged, and the serum was aspirated for the detection of biochemical markers. The liver was excised, weighed, and fixed in 10% formalin.

### 2.3 Serum analysis, liver staining, and scoring of lesions

Serum levels of total bilirubin (TBIL), direct bilirubin (DBIL), alanine aminotransferase (ALT), and aspartate aminotransferase (AST) were measured by an automatic biochemical analyzer. Liver tissue was fixed, dehydrated, embedded in paraffin, sectioned, and stained by hematoxylin and eosin (HE). The scoring of liver lesions was performed according to the Deleve standard (DeLeve et al., 1999) and involved the evaluation of (1) endothelial damage of the central venule (CV); (2) coagulative necrosis of hepatocytes; (3) subendothelial hemorrhage of the CV; (4) sinusoidal hemorrhage; (5) subendothelial fibrosis of the CV; (6) adventitial fibrosis of the CV. Each of these criteria was assigned a score of 0 to 3 points. Mild fibrosis of the CV was considered as early liver damage, and moderate and severe fibrosis was considered as advanced liver damage. The total score for early liver damage was the sum of scores for criteria 1 and 2 and the highest of the scores for criteria 3 and 4. The total score for late liver damage was the sum of the score for criterion 1, the highest of the scores for criteria 3 and 4, and the highest of the scores for criteria 5 and 6. The pathological scoring was performed by two independent pathologists who were blinded to the group assignments.

### 2.4 Analysis of microbial community composition

Fecal DNA was extracted using the DNA Kit (Omega Bio-tek, Norcross) according to the manufacturer's instructions. The quality of DNA samples was assessed by 1% agarose gel electrophoresis and spectrophotometry (260/280 nm optical density ratio). The bacterial 16S rDNA V3-V4 region was selected for PCR amplification, and the primers were 5′-ACTCCTACGGGAGGCAGCAG3′ (forward) and 5′-GGACTACNNGGGTATCTAAT3′ (reverse). PCR reaction mixture included 12.5 μl of 2 × Taq PCR MasterMix, 3 μl of BSA (2 ng/μl), 2 μl of primers (5 μM), 2 μl of template DNA, and 5.5 μl ddH_2_O. The amplification followed the steps: pre-denaturation at 95 °C for 5 min; 32 cycles of denaturation at 95 °C for 45 s, annealing at 55 °C for 50 s, and extension at 72 °C for 45 s; and the final extension at 72 °C for 10 min. The amplicons were separated by 2% agarose gel electrophoresis and recovered using the AxyPrep DNA Gel Extraction Kit (Axygen Biosciences). Paired-end sequencing was performed on the Illumina MiSeq PE300 platform, and the output data were filtered and spliced utilizing the QIIME (v1.8.0) software to remove raw reads with chimeras, scores lower than 20, base ambiguity, primers mismatch, and sequencing lengths shorter than 150 bp. Subsequently, the sequence information was clustered into OTU (Operational Taxonomic Units) with a similarity of 97%. For each OTU, the species were identified by comparison with the Silva138 16S rRNA database. The alpha diversity analysis was performed using the Mothur software (version 1.31.2). The UniFrac algorithm, which uses phylogenetic information, was employed to evaluate community differences between samples and to perform the beta diversity analysis. Principal coordinate analysis (PCoA) was calculated according to the UniFrac distance using a weighted algorithm. Linear discriminant analysis (LDA) effect size (LEfSe) was performed to determine the biological correlation and statistical significance in the relative abundances of the microbiota using an alpha value of 0.05 and an LDA threshold of 2.0.

### 2.5 Statistical analysis

Statistical calculations were conducted using the SPSS 26.0 software and GraphPad Prism (version 6.0). The Student's t-test was used to compare data with normal distribution, and Mann-Whitney U-test was used if a non-normal distribution was present. All statistical tests were two-tailed, and P-values < 0.05 were considered significant.

## 3 Results

### 3.1 Characterization of liver damage in the GS group

**Figure 1:**
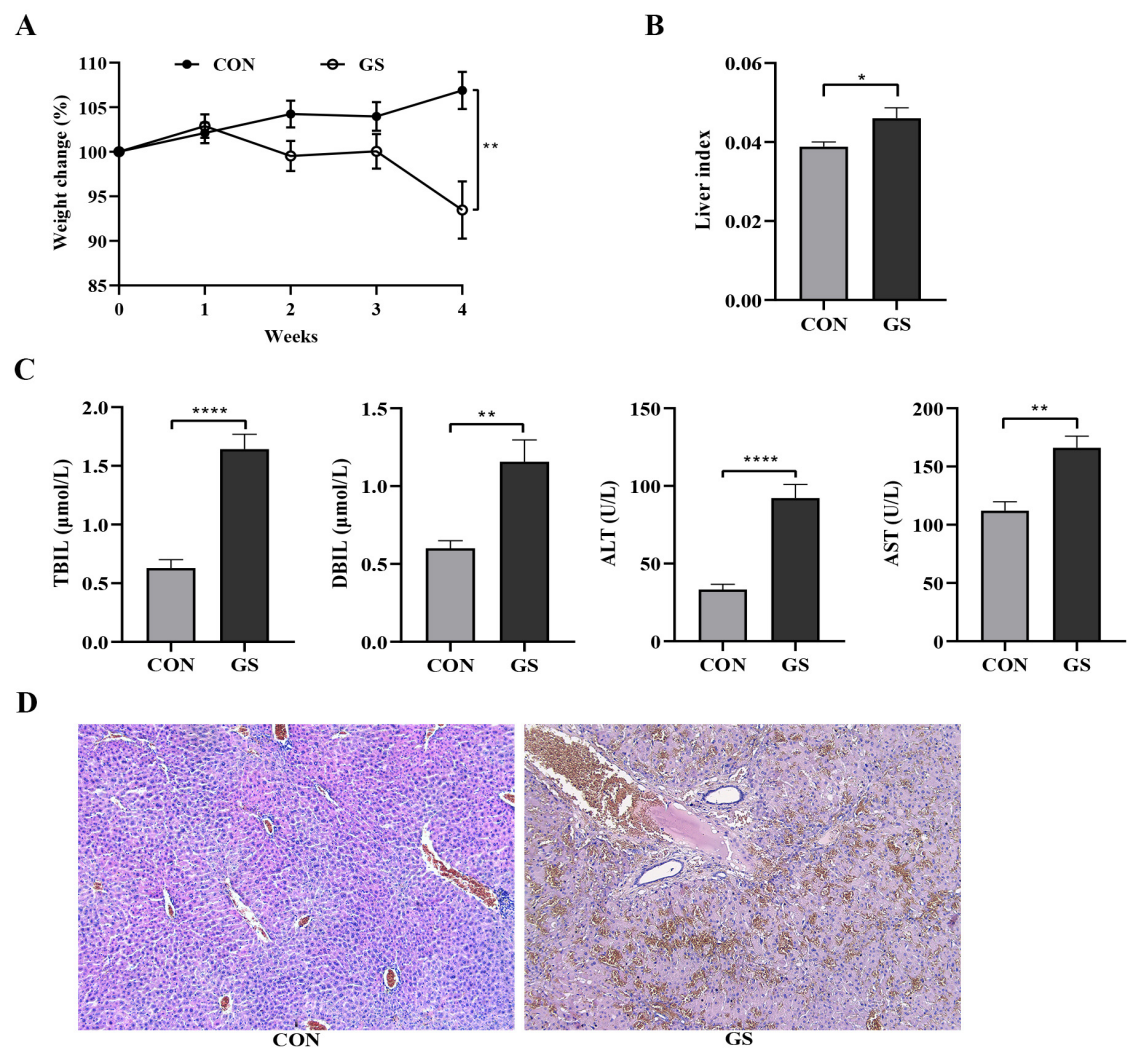
Effects of *Gynura Segetum* (GS) on liver function and histology. **(A)** Changes in body weight of mice after GS treatment. **(B)** Liver index (liver weight/body weight) of mice in the GS and control groups. **(C)** Serum levels of total bilirubin (TBIL), direct bilirubin (DBIL), alanine aminotransferase (ALT), and aspartate aminotransferase (AST) in GS-treated mice compared to control. **(D)** Hematoxylin and eosin (HE) stained liver sections in control and GS-treated mice. Images show liver damage including sinusoidal expansion, endothelial injury, and necrosis. Data are presented as the mean ± SEM. Significant differences are indicated as *p < 0.05.

To determine the role of intestinal flora in liver damage, mice were treated daily with GS decoction for 28 days, in the presence or absence of additional interventions described under Methods. The GS group was characterized by sparse hair, decreased motor activity, and increased abdominal girth compared to the control group. Control mice exhibited steady weight gain, while the animals in the GS group began to lose weight after the first week of treatment (Figure 1A). The liver index (liver weight/body weight) differed between the GS and control groups (Figure 1B). Mice receiving GS displayed a significant increase in TBIL, DBIL, ALT and AST in comparison with control animals (Figure 1C). Immediately upon harvesting, livers in the GS group were congested, swollen, and dark red. HE staining showed that the structure of hepatic lobules was intact in control mice but was disrupted in the GS group. Liver damage in GS-treated mice included different degrees of hepatic sinus expansion, endothelial cell injury, subendothelial hemorrhage, and hepatocyte necrosis (Figure 1D).

### 3.2 Changes in the gut microbiota structure during 4 weeks of GS administration

**Figure 2:**
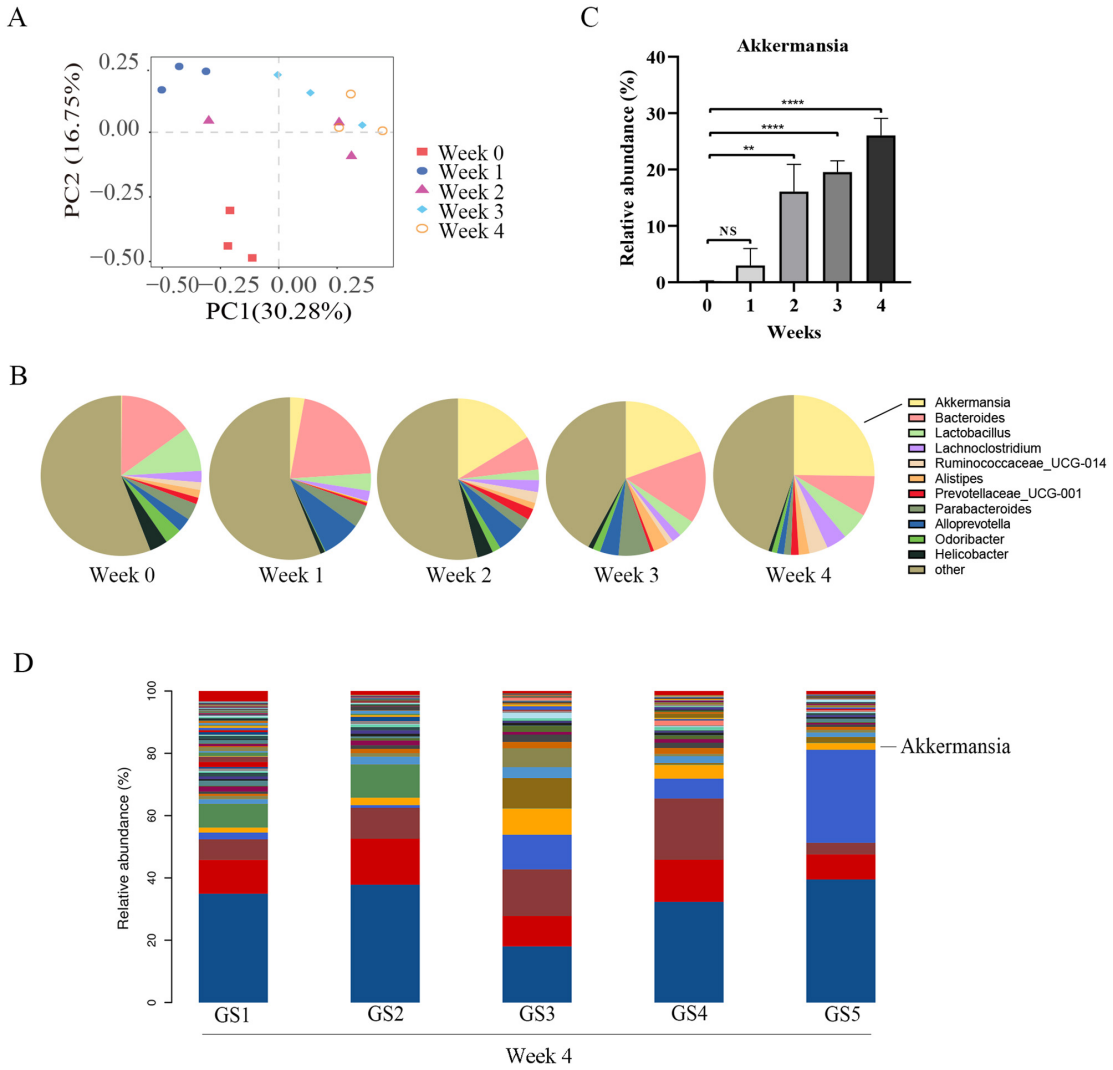
Impact of GS on Gut Microbiota Composition. **(A)** Principal component analysis (PCA) showing the differences in gut microbiota composition across different time points of GS treatment. **(B)** Pie charts depicting the composition of intestinal bacteria at the genus level in mice treated with GS. **(C)** Bar graph showing the relative abundance of *Akkermansia* in the feces of GS-treated mice. **(D)** Relative abundance of *Akkermansia* in the peritoneal fluid of GS-treated mice.

Previous studies have shown that HMs can generate pathogenic or therapeutic effects by interfering with bacterial metabolism, but whether GS can influence intestinal bacteria remains unknown. Therefore, we examined the composition of intestinal flora after GS-induced liver injury. Microbial sequences were obtained by high-throughput sequencing of 16S rRNA, and sequences with 97% similarity were clustered to form OTU, followed by species annotation. Principal coordinates analysis (PCoA) of beta-diversity showed that fecal samples from mice clustered into distinct groups at different time points, indicating that the microbial community had changed. For example, GS0 and GS4 were in different quadrants (Figure 2A). Pie charts in Figure 2B show the main species at the genus level at each week of the experiment. The dominant genera were *Bacteroides, Akkermansia, Lactobacillus, Lachnoclostridium, Ruminococcaceae_UCG-014, Alistipes, Prevotellaceae_UCG-001, Parabacteroides, Alloprevotella, Odoribacter, Helicobacter* and others, including many unidentified genera. After 4 weeks of treatment, the relative abundance of *Bacteroides* and *Lactobacillus* was reduced, while the abundance of *Akkermansia* increased. Other genera fluctuated with GS intake over time. The relative abundance of *Akkermansia* was 0.2% at the baseline, but increased 100-fold over 4 weeks of GS administration, reaching more than 20% (Figure 2C). Previous publications have found the translocated bacteria in lymph nodes, tumors, blood, and serous effusions that were originally considered sterile. The bacterial composition of peritoneal fluid was determined in mice treated with GS decoction. The spectrum of peritoneal flora was similar to that of the intestinal flora at the level of phyla and included *Firmicutes, Bacteroidetes, Proteobacteria, Actinobacteria, Verrucomicrobia*, among others. Notably, the proportion of *Akkermansia* was low in the ascitic fluid (Figure 2D), but greater than 20% in the feces.

### 3.3 Hepatic lesions in the GS, FMT, and Abx groups

**Figure 3:**
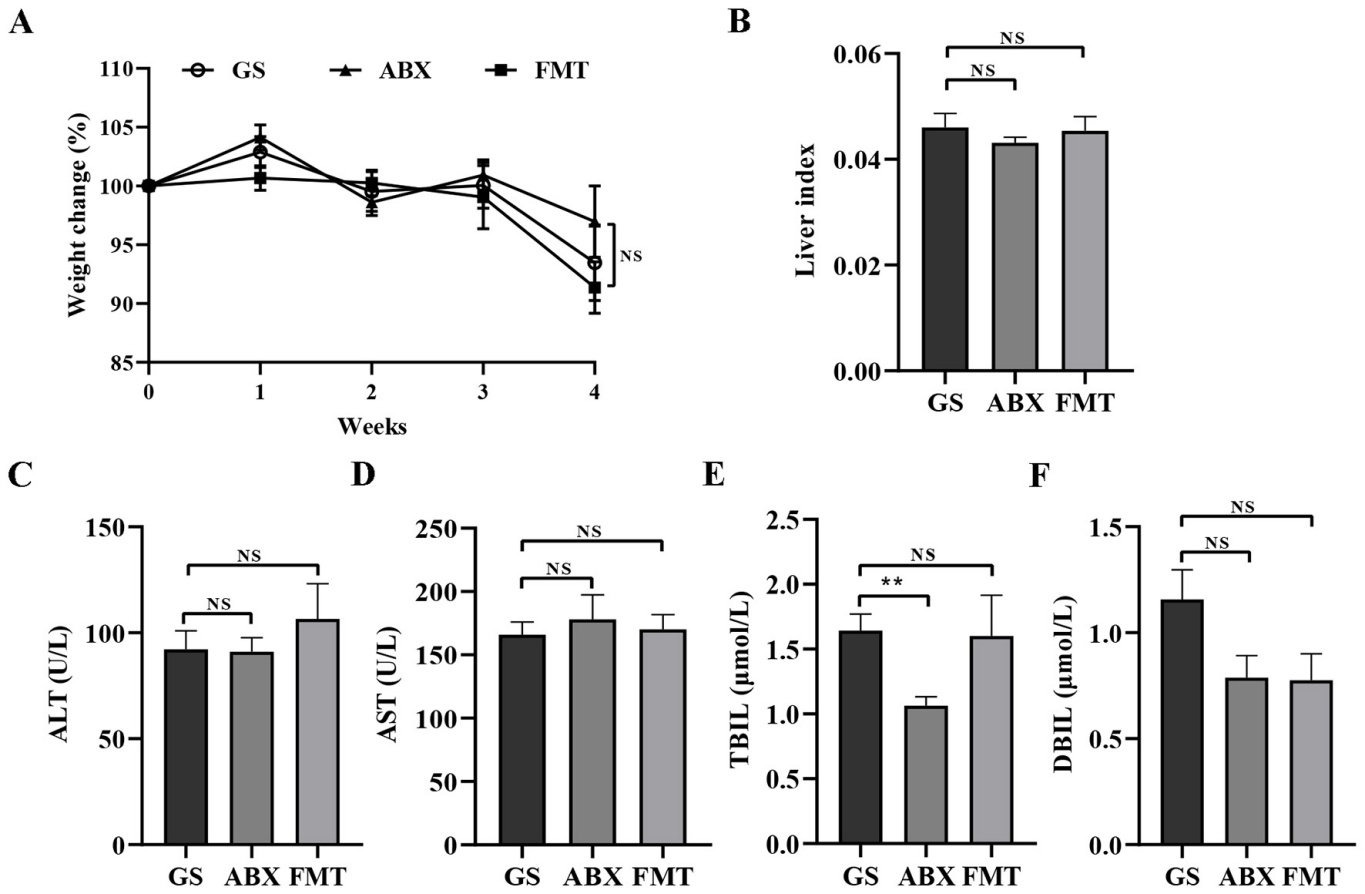
Effects of GS, antibiotic treatment (Abx), and fecal microbiota Transplantation (FMT) on Liver Function and Body Condition. **(A)** Body weight changes in the Abx, FMT and the GS groups. **(B)** Liver index (liver weight/body weight) of mice in the GS, Abx, and FMT groups. **(C-F)** Serum levels of ALT, AST, TBILand DBIL in the Abx, FMT and the GS groups. Data are presented as the mean ± SEM. Significant differences are indicated as *p < 0.05.

Antibiotics are used to treat infectious diseases, but, at the same time, they perturb the intestinal flora. FMT restores intestinal flora and can even treat diseases. The results indicated that 4 weeks of FMT or Abx treatment failed to reverse the symptoms induced by GS—including poor appetite, sparse hair, lethargy, reduced mobility, and weight loss (Figure 3A). Moreover, the liver index and the plasma concentrations of ALT and AST, TBIL, and DBIL were not statistically different between the GS and the FMT or Abx groups (Figures 3B-F). Consistent with the impaired liver function, the mice exhibited an increased liver pathology score, which was associated with CV endothelial damage, hepatic sinus congestion, and inflammation.

### 3.4 Comparison of gut microbiota between the GS and the FMT or Abx groups

**Figure 4:**
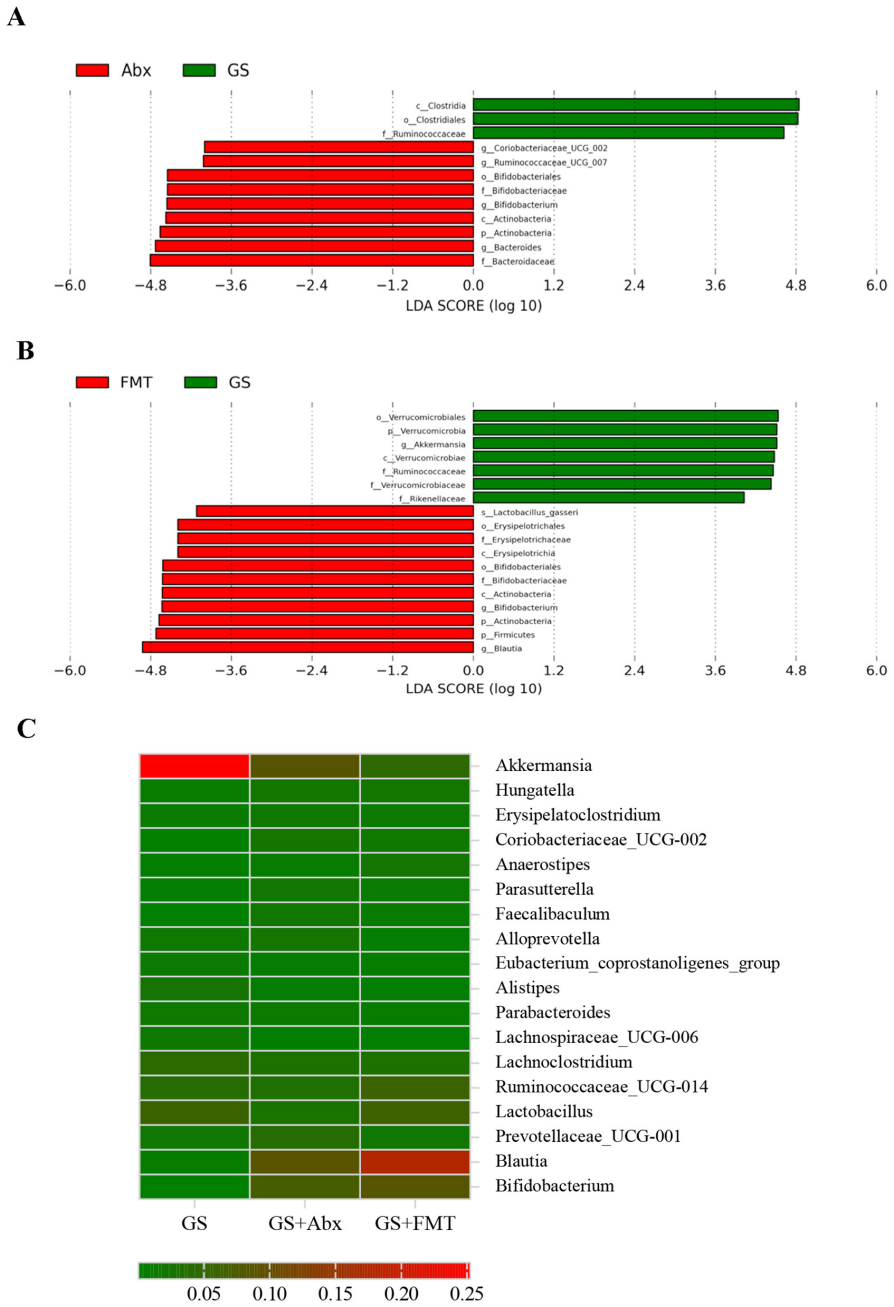
Alteration of gut microbiota by GS, Abx, and FMT. **(A)** Linear discriminant analysis (LDA) scores of the gut microbiota in GS and Abx-treated mice. **(B)** LDA scores of the gut microbiota in GS and FMT-treated mice, with an LDA threshold of 2. (**C**) Heatmap showing the relative abundance of dominant genera in the gut microbiota of GS, Abx, and FMT groups.

The results of the comparative analysis of gut microbiota among the experimental groups are illustrated in Figure 4. The LEfSe analysis enables a comparison of subgroups between and within groups to find species with significant differences in abundance. The results are displayed with an LDA distribution histogram. In comparison with mice treated with GS, the administration of Abx increased the relative abundance of the *Bacteroides* genus, a member of the *Bacteroidaceae* family (Figure 4A). In comparison with the GS group, FMT significantly increased the abundance of the *Bifidobacterium* genus (phylum Actinobacteria), as well as the *Blautia* genus (order Clostridiales) (Figure 4B). The heatmap depicts genus-level relative abundances of dominant taxa. Darker color indicates higher abundance. Figure 4C lists the top 18 genera, the abundance of which was noticeably different among the experimental groups. For example, the *Akkermansia* genus was more abundant, while the abundances of the *Blautia* and *Bifidobacterium* genera were significantly lower, or even non-detectable, in the GS group compared to the FMT and Abx groups. The relative abundance of the *Verrucomicrobi*a phylum, including the only representative genus *Akkermansia*, was evidently higher in the gut microbiota of mice in the GS group.

### 3.5 Modifications in gut microbiota induced by GS extracts

**Figure 5:**
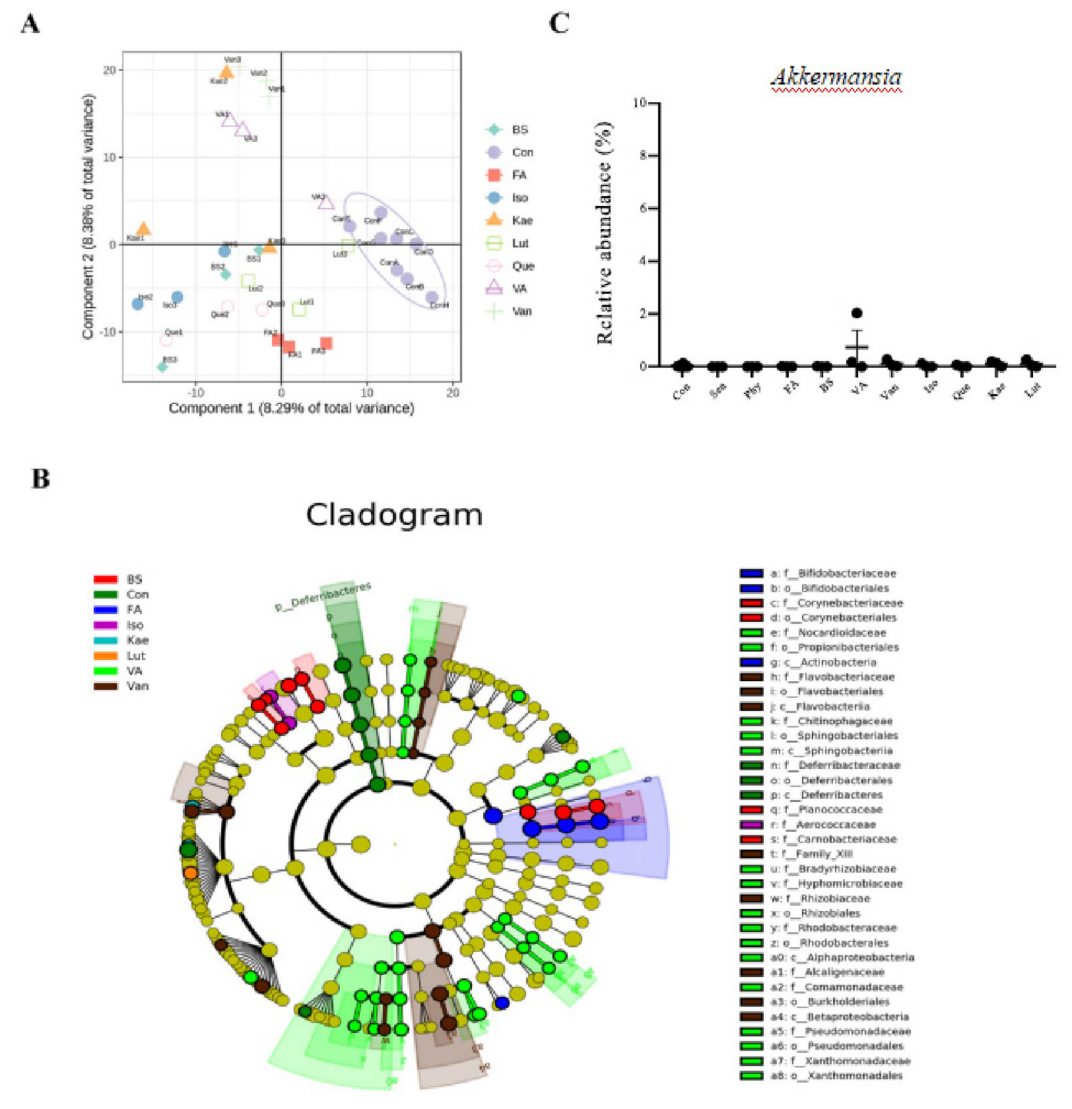
Modifications in gut microbiota by GS extracts. **(A)** Principal coordinate analysis (PCoA) showing microbiota differences in control (Con), Senecionine (Sen), Seneciphylline (Phy), Ferulic Acid (FA), Beta-sitosterol (BS), Vanillic Acid (VA), Vanillin (Van), Isorhamnetin (Iso), Quercetin (Que), Kaempferol (Kae), and Luteolin (Lut) treatment groups after 4 weeks. **(B)** Cladogram generated by Linear Discriminant Analysis (LEfSe) showing enriched taxa in the feces after 4 weeks of treatment. **(C)** Bar graph showing the relative abundance of the *Akkermansia* genus in the feces after 4 weeks of oral gavage with GS extracts.

The composition of gut microbiota was analyzed to determine the impact of specific components extracted from the GS decoction. The abundance of the *Akkermansia* genus did not increase significantly after 4 weeks of oral gavage with Sen, Phy, FA, BS, VA, Van, Iso, Que, Kae, and Lut, in comparison with and the Con group (Figure 5).

## 4 Discussion

In this study, we explored the effects of GS decoction on the gut microbiota of mice. GS-induced hepatotoxicity was evident from biochemical markers and liver pathology, and we observed significant fluctuations in the intestinal microbiota. “Interestingly, GS had a similar impact on liver function in mice pretreated with antibiotics or in those receiving FMT, suggesting that alterations in the gut microbiota neither worsened nor alleviated the liver damage.

After a 4-week gavage with the GS decoction, the abundance of the phylum *Verrucomicrobia* significantly increased, becoming comparable to *Bacteroidetes* and *Firmicutes*. The species *Akkermansia muciniphila* is the first member of the genus *Akkermansia* and the main representative of the phylum *Verrucomicrobia*. *A. muciniphila* inhabits the outer layer of the intestinal mucus, where it degrades mucin and produces short-chain fatty acids. *A. muciniphila* contributes to energy metabolism, maintenance of mucosal integrity, immune regulation, and cancer treatment (Rodrigues et al., 2022). In vitro and in vivo experiments have confirmed that the absence of *A. muciniphila* is a primary feature of metabolic and inflammatory diseases, and supplementing with live or pasteurized bacteria, or *A. muciniphila* extracellular vesicles (AmEVs), can reverse obesity, improve glucose tolerance and insulin resistance (Cani et al., 2022), reduce epithelial permeability, and thicken the mucus layer. *A. muciniphila* is regarded as the next-generation probiotic.

Although *A. muciniphila* is considered beneficial in various contexts, it has also been implicated in disease pathogenesis. In our study, the proportion of *Akkermansia* was found to be increased in mice with GS-induced hepatic injury. Analysis of a NAFLD patient cohort by Loomba et al. (2019) revealed a positive correlation between *A. muciniphila* abundance and hepatic fibrosis severity, potentially mediated through TLR4 pathway activation that promotes hepatic stellate cell activation. Consistent with this, Qiu et al. reported that Crohn's disease patients during active phases exhibited significantly elevated intestinal *A. muciniphila* levels, which positively correlated with inflammatory markers (calprotectin and CRP) (Qiu et al., 2022). Notably, high-dose administration in animal models may induce excessive mucus degradation (Chen et al., 2021). Consequently, in individuals and mice with impaired mucus secretion, over-colonization by *A. muciniphila* could increase intestinal permeability. Supporting this, case reports indicated its role as an opportunistic pathogen capable of causing bacteremia in immunocompromised patients (Sarvari et al., 2020). These findings collectively suggest that detrimental effects of *A. muciniphila* predominantly manifest in individuals with pre-existing pathological conditions (e.g., IBD or immunodeficiency) or under specific dietary regimens.

By employing an in vivo model mimicking the human colonic environment, it was possible to demonstrate that the HM therapy is effective in mice with healthy gut microbes or after FMT, while the efficacy was lost in sterile mice (Chen et al., 2016). In our study, the administration of Sen, Phy, FA, BS, VA, Van, Iso, Que, Kae, and Lut did not increase the abundance of *Akkermansia*. However, other studies have shown that the treatment of mice with Iso, Que, Van, and Kae can improve obesity and metabolism, and this effect is accompanied by an increased abundance of *Akkermansia* (Guo et al., 2018; Wang et al., 2020; Bosch-Sierra et al., 2021). The discrepancy between these findings may be due to differences in species, mutual interference, clinical transformation bottlenecks, and even unknown ingredients dissolved in the GS decoction. The chemical components of HM were separated into multiple categories, such as carbohydrates and non-carbohydrate small molecules since various types of chemicals contained in HM can interact with the microbiota through different mechanisms. Although modern spectral identification techniques have characterized many ingredients of the GS decoction, polar compounds, such as polysaccharides, are difficult to identify. However, specific mechanisms by which GS perturbs the intestinal flora, and the causal links to hepatotoxicity, warrant further investigation.

Our study found that the abundance of the *Akkermansia* genus was decreased in the Abx group in comparison with the GS group. Previous studies have shown that vancomycin treatment of *C. difficile* infection in children made *A. muciniphila* the most abundant species (Aragon et al., 2023), which is inconsistent with our findings. In contrast, treatment with imipenem for 10 days in an intensive care unit (ICU) setting increased the abundance of *A. muciniphila* to 84.6%. However, in vitro experiments have shown that *A. muciniphila* is sensitive to imipenem, piperacillin/tazobactam, and doxycycline (Dubourg et al., 2013). This discrepancy was most likely due to population differences, individual variation, and experimental environment.

The genus *Ruminococcaceae* is considered as a beneficial, autochthonous taxon capable of fermenting fibers to produce short-chain fatty acids (SCFAs) and exhibiting anti-inflammatory activity in the gut. A decrease in the number of *Ruminococcaceae* was observed in nonalcoholic fatty liver disease (NAFLD) (Hui et al., 2023). In contrast to these reports, we noted an increase in *Ruminococcaceae_UCG-014* in mice with GS-induced hepatic lesions, and Ponziani demonstrated similar changes in patients with cirrhosis and hepatocellular carcinoma (HCC) compared to those without (Ponziani et al., 2019). Future research should focus on elucidating strain-specific differences and establishing causal relationships. *Bifidobacteria* can synthesize glycosylhydrolases (GHs) that can degrade complex glycans, glycoproteins, mucin, and human milk oligosaccharides. The abundance of *Bifidobacteria* was decreased in animals fed with a high-fat diet or mice with non-alcoholic fatty liver and was inversely correlated with inflammatory factors. Previous studies demonstrated that the abundance of *Bifidobacteria* was stably increased after the administration of microbiota transfer therapy in patients with autism spectrum disorder (Abujamel et al., 2022), and gastrointestinal symptoms continued to be improved at follow-up. In agreement with the above publications, we documented an increase in *Bifidobacteria* in the FMT group when compared to the GS group.

Because *Blautia* can convert carbohydrates and proteins into acetic acid, its depletion contributes to the pathogenesis and progression of hepatic disorders through gut-liver axis mechanisms. Pretreatment with *B. producta* strains D4 and DSM2950 significantly reduced liver function biomarkers and inflammatory cytokine levels in mice with acute liver injury (ALI) (Mao et al., 2023). The low or non-detectable level of *Blautia* in GS-treated mice may be associated with exhaustion or the competition for the same substrates by *Akkermansia*. *Blautia* (Liu et al., 2021), a genus encompassing multiple species, has been implicated in inflammatory processes. For instance, the *Blautia coccoides* species has been shown to stimulate the secretion of inflammatory factors such as TNF-α. Future investigations should extend to species- and strain-level analyses.

The present study elucidated the effect of GS on the intestinal flora and demonstrated a remarkable increase in the abundance of the *Akkermansia* genus. Since *Akkermansia* are anaerobic, the in vitro growth medium requires animal-derived culture, which leads to its limited clinical application. GS can directly increase the beneficial *Akkermansia* genus for therapeutic purposes if the manipulation of the microbial ecology could be optimized precisely and according to the individual needs of the patient. This possibility can materialize with the discovery of unknown active components of the GS decoction. However, the augmentation of *Akkermansia* did not protect mice from GS-induced liver injury. This result reinforces the notion that probiotic treatment alone may not achieve disease resolution, but may serve only as a supplemental intervention. Furthermore, we demonstrated that liver injury in the GS-fed mice was similar with or without the intervention targeting bacterial flora, such as the use of antibiotics or FMT. Thus, we propose that therapies that regulate intestinal flora require large, long-term, multi-regional studies, and clinical trials involving diverse races and ethnicities. Future research should focus on isolating and identifying the active components in GS that promote the growth of *Akkermansia*, elucidating the mechanisms through which GS regulates *Akkermansia* and its metabolites.

## 5 Conclusion

Liver damage caused by GS decoction was accompanied by the perturbation of the intestinal flora, particularly by the persistently increased abundance of *Akkermansia*. Oral gavage with GS, combined with FMT or Abx, destabilized the gut microbiota but did not protect against GS-induced liver injury.

## Data Availability

The data presented in the study are deposited in the BioProject database, accession number PRJNA1357622.
